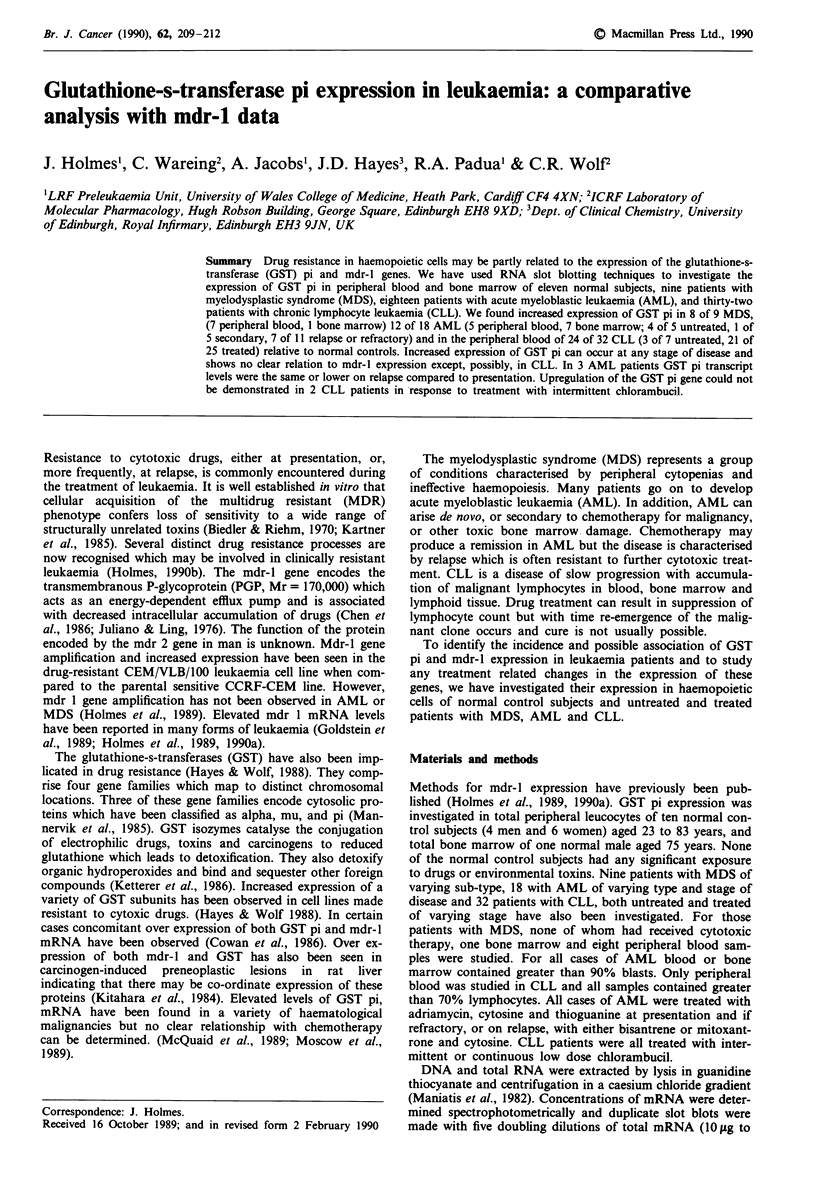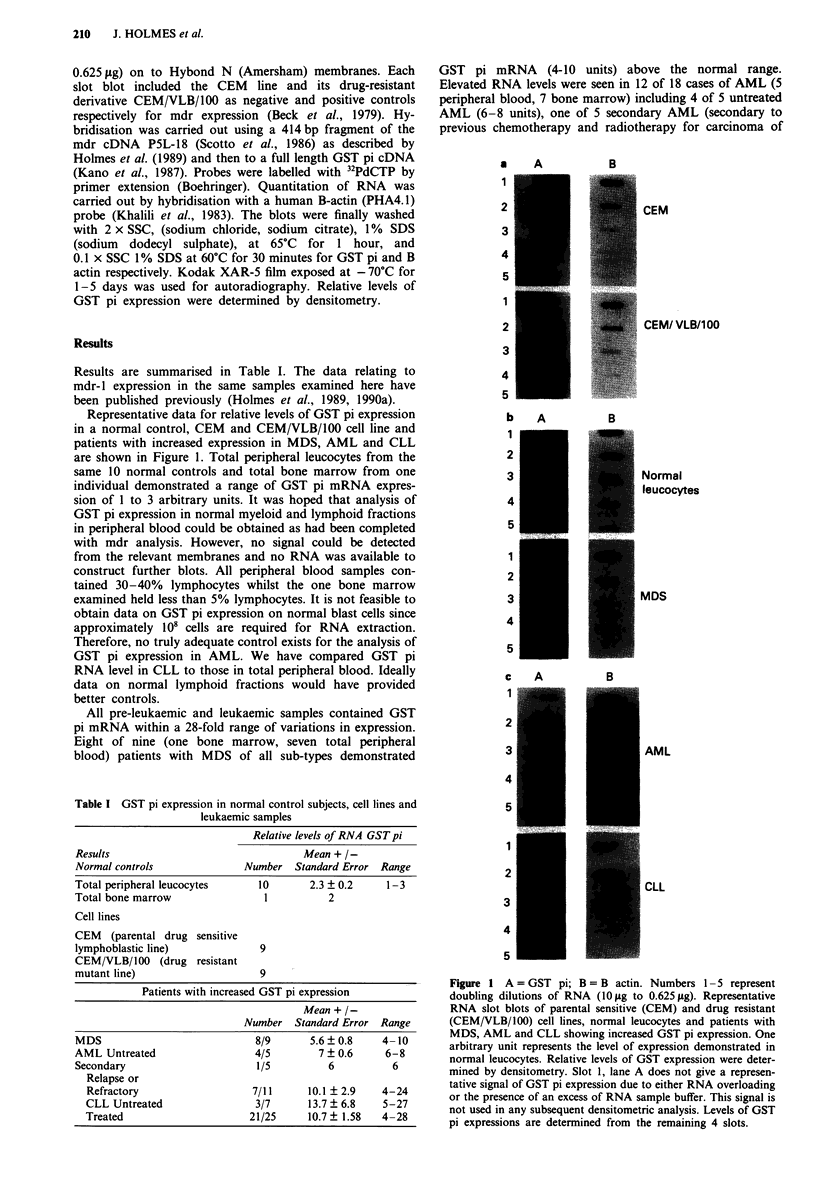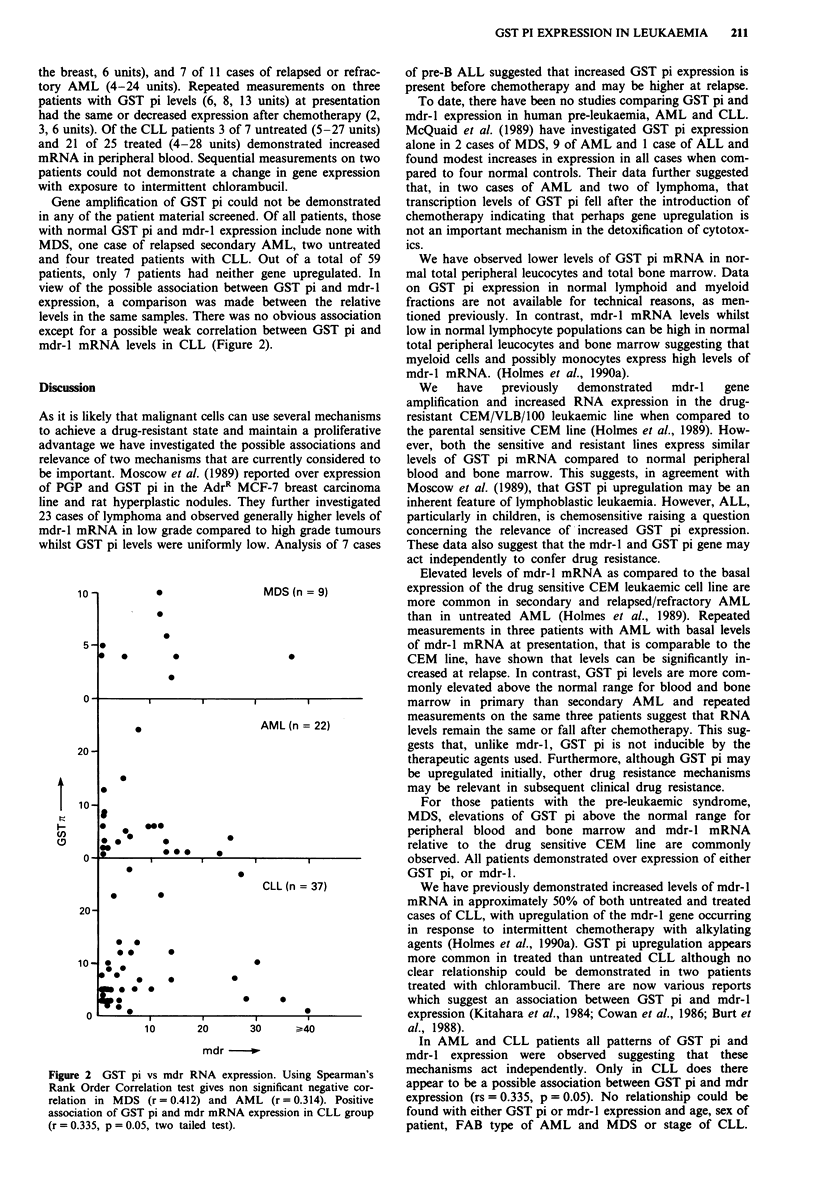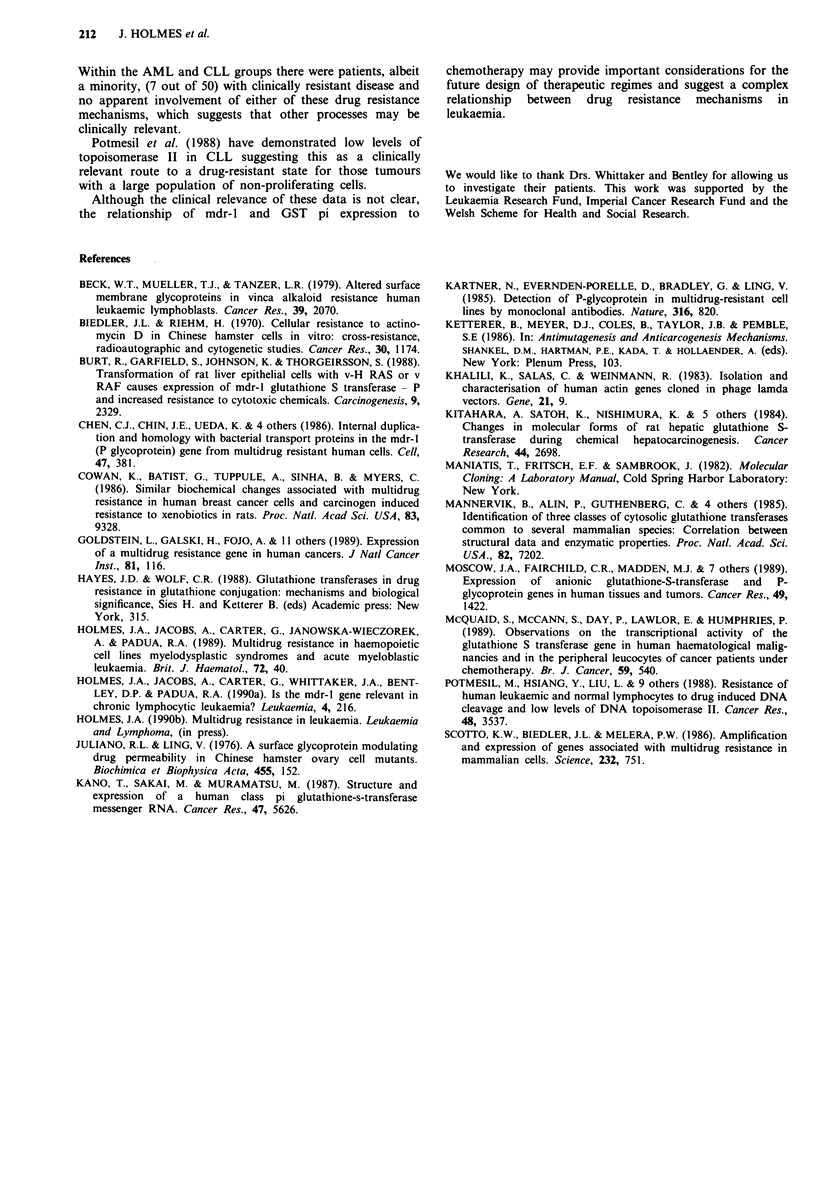# Glutathione-s-transferase pi expression in leukaemia: a comparative analysis with mdr-1 data.

**DOI:** 10.1038/bjc.1990.262

**Published:** 1990-08

**Authors:** J. Holmes, C. Wareing, A. Jacobs, J. D. Hayes, R. A. Padua, C. R. Wolf

**Affiliations:** LRF Preleukaemia Unit, University of Wales College of Medicine, Heath Park, Cardiff, UK.

## Abstract

**Images:**


Br. J. Cancer (1990), 62, 209-212 © Macmillan Press Ltd., 1990

### Glutathione-s-transferase pi expression in leukaemia: a comparative analysis with mdr-1 data

J. Holmes[1], C. Wareing[2], A. Jacobs[1], J.D. Hayes[3], R.A. Padua[1] & C.R. Wolf[2]

[1]*LRF Preleukaemia Unit, University of Wales College of Medicine, Heath Park, Cardiff CF4 4XN;* [2]*ICRF Laboratory of Molecular Pharmacology, Hugh Robson Building, George Square, Edinburgh EH8 9XD;* [3]*Dept. of Clinical Chemistry, University of Edinburgh, Royal Infirmary, Edinburgh EH3 9JN, UK*

**Summary** Drug resistance in haemopoietic cells may be partly related to the expression of the glutathione-s-transferase (GST) pi and mdr-1 genes. We have used RNA slot blotting techniques to investigate the expression of GST pi in peripheral blood and bone marrow of eleven normal subjects, nine patients with myelodysplastic syndrome (MDS), eighteen patients with acute myeloblastic leukaemia (AML), and thirty-two patients with chronic lymphocyte leukaemia (CLL). We found increased expression of GST pi in 8 of 9 MDS, (7 peripheral blood, 1 bone marrow) 12 of 18 AML (5 peripheral blood, 7 bone marrow; 4 of 5 untreated, 1 of 5 secondary, 7 of 11 relapse or refractory) and in the peripheral blood of 24 of 32 CLL (3 of 7 untreated, 21 of 25 treated) relative to normal controls. Increased expression of GST pi can occur at any stage of disease and shows no clear relation to mdr-1 expression except, possibly, in CLL. In 3 AML patients GST pi transcript levels were the same or lower on relapse compared to presentation. Upregulation of the GST pi gene could not be demonstrated in 2 CLL patients in response to treatment with intermittent chlorambucil.

Resistance to cytotoxic drugs, either at presentation, or, more frequently, at relapse, is commonly encountered during the treatment of leukaemia. It is well established *in vitro* that cellular acquisition of the multidrug resistant (MDR) phenotype confers loss of sensitivity to a wide range of structurally unrelated toxins (Biedler & Riehm, 1970; Kartner *et al.*, 1985). Several distinct drug resistance processes are now recognised which may be involved in clinically resistant leukaemia (Holmes, 1990b). The mdr-1 gene encodes the transmembranous P-glycoprotein (PGP, Mr = 170,000) which acts as an energy-dependent efflux pump and is associated with decreased intracellular accumulation of drugs (Chen *et al.*, 1986; Juliano & Ling, 1976). The function of the protein encoded by the mdr 2 gene in man is unknown. Mdr-1 gene amplification and increased expression have been seen in the drug-resistant CEM/VLB/100 leukaemia cell line when compared to the parental sensitive CCRF-CEM line. However, mdr 1 gene amplification has not been observed in AML or MDS (Holmes *et al.*, 1989). Elevated mdr 1 mRNA levels have been reported in many forms of leukaemia (Goldstein *et al.*, 1989; Holmes *et al.*, 1989, 1990a).

The glutathione-s-transferases (GST) have also been implicated in drug resistance (Hayes & Wolf, 1988). They comprise four gene families which map to distinct chromosomal locations. Three of these gene families encode cytosolic proteins which have been classified as alpha, mu, and pi (Mannervik *et al.*, 1985). GST isozymes catalyse the conjugation of electrophilic drugs, toxins and carcinogens to reduced glutathione which leads to detoxification. They also detoxify organic hydroperoxides and bind and sequester other foreign compounds (Ketterer *et al.*, 1986). Increased expression of a variety of GST subunits has been observed in cell lines made resistant to cytoxic drugs. (Hayes & Wolf 1988). In certain cases concomitant over expression of both GST pi and mdr-1 mRNA have been observed (Cowan *et al.*, 1986). Over expression of both mdr-1 and GST has also been seen in carcinogen-induced preneoplastic lesions in rat liver indicating that there may be co-ordinate expression of these proteins (Kitahara *et al.*, 1984). Elevated levels of GST pi, mRNA have been found in a variety of haematological malignancies but no clear relationship with chemotherapy can be determined. (McQuaid *et al.*, 1989; Moscow *et al.*, 1989).

The myelodysplastic syndrome (MDS) represents a group of conditions characterised by peripheral cytopenias and ineffective haemopoiesis. Many patients go on to develop acute myeloblastic leukaemia (AML). In addition, AML can arise *de novo*, or secondary to chemotherapy for malignancy, or other toxic bone marrow damage. Chemotherapy may produce a remission in AML but the disease is characterised by relapse which is often resistant to further cytotoxic treatment. CLL is a disease of slow progression with accumulation of malignant lymphocytes in blood, bone marrow and lymphoid tissue. Drug treatment can result in suppression of lymphocyte count but with time re-emergence of the malignant clone occurs and cure is not usually possible.

To identify the incidence and possible association of GST pi and mdr-1 expression in leukaemia patients and to study any treatment related changes in the expression of these genes, we have investigated their expression in haemopoietic cells of normal control subjects and untreated and treated patients with MDS, AML and CLL.

### Materials and methods

Methods for mdr-1 expression have previously been published (Holmes *et al.*, 1989, 1990a). GST pi expression was investigated in total peripheral leucocytes of ten normal control subjects (4 men and 6 women) aged 23 to 83 years, and total bone marrow of one normal male aged 75 years. None of the normal control subjects had any significant exposure to drugs or environmental toxins. Nine patients with MDS of varying sub-type, 18 with AML of varying type and stage of disease and 32 patients with CLL, both untreated and treated of varying stage have also been investigated. For those patients with MDS, none of whom had received cytotoxic therapy, one bone marrow and eight peripheral blood samples were studied. For all cases of AML blood or bone marrow contained greater than 90% blasts. Only peripheral blood was studied in CLL and all samples contained greater than 70% lymphocytes. All cases of AML were treated with adriamycin, cytosine and thioguanine at presentation and if refractory, or on relapse, with either bisantrene or mitoxantrone and cytosine. CLL patients were all treated with intermittent or continuous low dose chlorambucil.

DNA and total RNA were extracted by lysis in guanidine thiocyanate and centrifugation in a caesium chloride gradient (Maniatis *et al.*, 1982). Concentrations of mRNA were determined spectrophotometrically and duplicate slot blots were made with five doubling dilutions of total mRNA (10 $\mu$g to

Correspondence: J. Holmes.
Received 16 October 1989; and in revised form 2 February 1990

0.625 µg) on to Hybond N (Amersham) membranes. Each slot blot included the CEM line and its drug-resistant derivative CEM/VLB/100 as negative and positive controls respectively for mdr expression (Beck *et al.*, 1979). Hybridisation was carried out using a 414 bp fragment of the mdr cDNA P5L-18 (Scotto *et al.*, 1986) as described by Holmes *et al.* (1989) and then to a full length GST pi cDNA (Kano *et al.*, 1987). Probes were labelled with $^{32}$PdCTP by primer extension (Boehringer). Quantitation of RNA was carried out by hybridisation with a human B-actin (PHA4.1) probe (Khalili *et al.*, 1983). The blots were finally washed with 2 × SSC, (sodium chloride, sodium citrate), 1% SDS (sodium dodecyl sulphate), at 65°C for 1 hour, and 0.1 × SSC 1% SDS at 60°C for 30 minutes for GST pi and B actin respectively. Kodak XAR-5 film exposed at −70°C for 1–5 days was used for autoradiography. Relative levels of GST pi expression were determined by densitometry.

### Results

Results are summarised in Table I. The data relating to mdr-1 expression in the same samples examined here have been published previously (Holmes *et al.*, 1989, 1990a).

Representative data for relative levels of GST pi expression in a normal control, CEM and CEM/VLB/100 cell line and patients with increased expression in MDS, AML and CLL are shown in Figure 1. Total peripheral leucocytes from the same 10 normal controls and total bone marrow from one individual demonstrated a range of GST pi mRNA expression of 1 to 3 arbitrary units. It was hoped that analysis of GST pi expression in normal myeloid and lymphoid fractions in peripheral blood could be obtained as had been completed with mdr analysis. However, no signal could be detected from the relevant membranes and no RNA was available to construct further blots. All peripheral blood samples contained 30–40% lymphocytes whilst the one bone marrow examined held less than 5% lymphocytes. It is not feasible to obtain data on GST pi expression on normal blast cells since approximately $10^8$ cells are required for RNA extraction. Therefore, no truly adequate control exists for the analysis of GST pi expression in AML. We have compared GST pi RNA level in CLL to those in total peripheral blood. Ideally data on normal lymphoid fractions would have provided better controls.

All pre-leukaemic and leukaemic samples contained GST pi mRNA within a 28-fold range of variations in expression. Eight of nine (one bone marrow, seven total peripheral blood) patients with MDS of all sub-types demonstrated GST pi mRNA (4-10 units) above the normal range. Elevated RNA levels were seen in 12 of 18 cases of AML (5 peripheral blood, 7 bone marrow) including 4 of 5 untreated AML (6–8 units), one of 5 secondary AML (secondary to previous chemotherapy and radiotherapy for carcinoma of

**Table I** GST pi expression in normal control subjects, cell lines and leukaemic samples

| | Relative levels of RNA GST pi | | |
|---|---|---|---|
| *Results* *Normal controls* | *Number* | *Mean + / − Standard Error* | *Range* |
| Total peripheral leucocytes | 10 | 2.3 ± 0.2 | 1–3 |
| Total bone marrow | 1 | 2 | |
| Cell lines | | | |
| CEM (parental drug sensitive lymphoblastic line) | 9 | | |
| CEM/VLB/100 (drug resistant mutant line) | 9 | | |
| Patients with increased GST pi expression | | | |
| | *Number* | *Mean + / − Standard Error* | *Range* |
| MDS | 8/9 | 5.6 ± 0.8 | 4–10 |
| AML Untreated | 4/5 | 7 ± 0.6 | 6–8 |
| Secondary | 1/5 | 6 | 6 |
| Relapse or Refractory | 7/11 | 10.1 ± 2.9 | 4–24 |
| CLL Untreated | 3/7 | 13.7 ± 6.8 | 5–27 |
| Treated | 21/25 | 10.7 ± 1.58 | 4–28 |

**Figure 1** A = GST pi; B = B actin. Numbers 1–5 represent doubling dilutions of RNA (10 µg to 0.625 µg). Representative RNA slot blots of parental sensitive (CEM) and drug resistant (CEM/VLB/100) cell lines, normal leucocytes and patients with MDS, AML and CLL showing increased GST pi expression. One arbitrary unit represents the level of expression demonstrated in normal leucocytes. Relative levels of GST expression were determined by densitometry. Slot 1, lane A does not give a representative signal of GST pi expression due to either RNA overloading or the presence of an excess of RNA sample buffer. This signal is not used in any subsequent densitometric analysis. Levels of GST pi expressions are determined from the remaining 4 slots.

the breast, 6 units), and 7 of 11 cases of relapsed or refractory AML (4–24 units). Repeated measurements on three patients with GST pi levels (6, 8, 13 units) at presentation had the same or decreased expression after chemotherapy (2, 3, 6 units). Of the CLL patients 3 of 7 untreated (5–27 units) and 21 of 25 treated (4–28 units) demonstrated increased mRNA in peripheral blood. Sequential measurements on two patients could not demonstrate a change in gene expression with exposure to intermittent chlorambucil.

Gene amplification of GST pi could not be demonstrated in any of the patient material screened. Of all patients, those with normal GST pi and mdr-1 expression include none with MDS, one case of relapsed secondary AML, two untreated and four treated patients with CLL. Out of a total of 59 patients, only 7 patients had neither gene upregulated. In view of the possible association between GST pi and mdr-1 expression, a comparison was made between the relative levels in the same samples. There was no obvious association except for a possible weak correlation between GST pi and mdr-1 mRNA levels in CLL (Figure 2).

### Discussion

As it is likely that malignant cells can use several mechanisms to achieve a drug-resistant state and maintain a proliferative advantage we have investigated the possible associations and relevance of two mechanisms that are currently considered to be important. Moscow *et al.* (1989) reported over expression of PGP and GST pi in the $Adr^R$ MCF-7 breast carcinoma line and rat hyperplastic nodules. They further investigated 23 cases of lymphoma and observed generally higher levels of mdr-1 mRNA in low grade compared to high grade tumours whilst GST pi levels were uniformly low. Analysis of 7 cases

**Figure 2** GST pi vs mdr RNA expression. Using Spearman's Rank Order Correlation test gives non significant negative correlation in MDS (r = 0.412) and AML (r = 0.314). Positive association of GST pi and mdr mRNA expression in CLL group (r = 0.335, p = 0.05, two tailed test).

of pre-B ALL suggested that increased GST pi expression is present before chemotherapy and may be higher at relapse.

To date, there have been no studies comparing GST pi and mdr-1 expression in human pre-leukaemia, AML and CLL. McQuaid *et al.* (1989) have investigated GST pi expression alone in 2 cases of MDS, 9 of AML and 1 case of ALL and found modest increases in expression in all cases when compared to four normal controls. Their data further suggested that, in two cases of AML and two of lymphoma, that transcription levels of GST pi fell after the introduction of chemotherapy indicating that perhaps gene upregulation is not an important mechanism in the detoxification of cytotoxics.

We have observed lower levels of GST pi mRNA in normal total peripheral leucocytes and total bone marrow. Data on GST pi expression in normal lymphoid and myeloid fractions are not available for technical reasons, as mentioned previously. In contrast, mdr-1 mRNA levels whilst low in normal lymphocyte populations can be high in normal total peripheral leucocytes and bone marrow suggesting that myeloid cells and possibly monocytes express high levels of mdr-1 mRNA. (Holmes *et al.*, 1990a).

We have previously demonstrated mdr-1 gene amplification and increased RNA expression in the drug-resistant CEM/VLB/100 leukaemic line when compared to the parental sensitive CEM line (Holmes *et al.*, 1989). However, both the sensitive and resistant lines express similar levels of GST pi mRNA compared to normal peripheral blood and bone marrow. This suggests, in agreement with Moscow *et al.* (1989), that GST pi upregulation may be an inherent feature of lymphoblastic leukaemia. However, ALL, particularly in children, is chemosensitive raising a question concerning the relevance of increased GST pi expression. These data also suggest that the mdr-1 and GST pi gene may act independently to confer drug resistance.

Elevated levels of mdr-1 mRNA as compared to the basal expression of the drug sensitive CEM leukaemic cell line are more common in secondary and relapsed/refractory AML than in untreated AML (Holmes *et al.*, 1989). Repeated measurements in three patients with AML with basal levels of mdr-1 mRNA at presentation, that is comparable to the CEM line, have shown that levels can be significantly increased at relapse. In contrast, GST pi levels are more commonly elevated above the normal range for blood and bone marrow in primary than secondary AML and repeated measurements on the same three patients suggest that RNA levels remain the same or fall after chemotherapy. This suggests that, unlike mdr-1, GST pi is not inducible by the therapeutic agents used. Furthermore, although GST pi may be upregulated initially, other drug resistance mechanisms may be relevant in subsequent clinical drug resistance.

For those patients with the pre-leukaemic syndrome, MDS, elevations of GST pi above the normal range for peripheral blood and bone marrow and mdr-1 mRNA relative to the drug sensitive CEM line are commonly observed. All patients demonstrated over expression of either GST pi, or mdr-1.

We have previously demonstrated increased levels of mdr-1 mRNA in approximately 50% of both untreated and treated cases of CLL, with upregulation of the mdr-1 gene occurring in response to intermittent chemotherapy with alkylating agents (Holmes *et al.*, 1990a). GST pi upregulation appears more common in treated than untreated CLL although no clear relationship could be demonstrated in two patients treated with chlorambucil. There are now various reports which suggest an association between GST pi and mdr-1 expression (Kitahara *et al.*, 1984; Cowan *et al.*, 1986; Burt *et al.*, 1988).

In AML and CLL patients all patterns of GST pi and mdr-1 expression were observed suggesting that these mechanisms act independently. Only in CLL does there appear to be a possible association between GST pi and mdr expression (rs = 0.335, p = 0.05). No relationship could be found with either GST pi or mdr-1 expression and age, sex of patient, FAB type of AML and MDS or stage of CLL.

Within the AML and CLL groups there were patients, albeit a minority, (7 out of 50) with clinically resistant disease and no apparent involvement of either of these drug resistance mechanisms, which suggests that other processes may be clinically relevant.

Potmesil *et al.* (1988) have demonstrated low levels of topoisomerase II in CLL suggesting this as a clinically relevant route to a drug-resistant state for those tumours with a large population of non-proliferating cells.

Although the clinical relevance of these data is not clear, the relationship of mdr-1 and GST pi expression to chemotherapy may provide important considerations for the future design of therapeutic regimes and suggest a complex relationship between drug resistance mechanisms in leukaemia.

We would like to thank Drs. Whittaker and Bentley for allowing us to investigate their patients. This work was supported by the Leukaemia Research Fund, Imperial Cancer Research Fund and the Welsh Scheme for Health and Social Research.

### References

BECK, W.T., MUELLER, T.J., & TANZER, L.R. (1979). Altered surface membrane glycoproteins in vinca alkaloid resistance human leukaemic lymphoblasts. *Cancer Res.*, **39**, 2070.

BIEDLER, J.L. & RIEHM, H. (1970). Cellular resistance to actinomycin D in Chinese hamster cells in vitro: cross-resistance, radioautographic and cytogenetic studies. *Cancer Res.*, **30**, 1174.

BURT, R., GARFIELD, S., JOHNSON, K. & THORGEIRSSON, S. (1988). Transformation of rat liver epithelial cells with v-H RAS or v RAF causes expression of mdr-1 glutathione S transferase – P and increased resistance to cytotoxic chemicals. *Carcinogenesis*, **9**, 2329.

CHEN, C.J., CHIN, J.E., UEDA, K. & 4 others (1986). Internal duplication and homology with bacterial transport proteins in the mdr-1 (P glycoprotein) gene from multidrug resistant human cells. *Cell*, **47**, 381.

COWAN, K., BATIST, G., TUPPULE, A., SINHA, B. & MYERS, C. (1986). Similar biochemical changes associated with multidrug resistance in human breast cancer cells and carcinogen induced resistance to xenobiotics in rats. *Proc. Natl. Acad Sci. USA*, **83**, 9328.

GOLDSTEIN, L., GALSKI, H., FOJO, A. & 11 others (1989). Expression of a multidrug resistance gene in human cancers. *J Natl Cancer Inst.*, **81**, 116.

HAYES, J.D. & WOLF, C.R. (1988). Glutathione transferases in drug resistance in glutathione conjugation: mechanisms and biological significance, Sies H. and Ketterer B. (eds) Academic press: New York, 315.

HOLMES, J.A., JACOBS, A., CARTER, G., JANOWSKA-WIECZOREK, A. & PADUA, R.A. (1989). Multidrug resistance in haemopoietic cell lines myelodysplastic syndromes and acute myeloblastic leukaemia. *Brit. J. Haematol.*, **72**, 40.

HOLMES, J.A., JACOBS, A., CARTER, G., WHITTAKER, J.A., BENTLEY, D.P. & PADUA, R.A. (1990a). Is the mdr-1 gene relevant in chronic lymphocytic leukaemia? *Leukaemia*, **4**, 216.

HOLMES, J.A. (1990b). Multidrug resistance in leukaemia. *Leukaemia and Lymphoma*, (in press).

JULIANO, R.L. & LING, V. (1976). A surface glycoprotein modulating drug permeability in Chinese hamster ovary cell mutants. *Biochimica et Biophysica Acta*, **455**, 152.

KANO, T., SAKAI, M. & MURAMATSU, M. (1987). Structure and expression of a human class pi glutathione-s-transferase messenger RNA. *Cancer Res.*, **47**, 5626.

KARTNER, N., EVERNDEN-PORELLE, D., BRADLEY, G. & LING, V. (1985). Detection of P-glycoprotein in multidrug-resistant cell lines by monoclonal antibodies. *Nature*, **316**, 820.

KETTERER, B., MEYER, D.J., COLES, B., TAYLOR, J.B. & PEMBLE, S.E (1986). In: *Antimutagenesis and Anticarcogenesis Mechanisms*. SHANKEL, D.M., HARTMAN, P.E., KADA, T. & HOLLAENDER, A. (eds). New York: Plenum Press, 103.

KHALILI, K., SALAS, C. & WEINMANN, R. (1983). Isolation and characterisation of human actin genes cloned in phage lamda vectors. *Gene*, **21**, 9.

KITAHARA, A. SATOH, K., NISHIMURA, K. & 5 others (1984). Changes in molecular forms of rat hepatic glutathione S-transferase during chemical hepatocarcinogenesis. *Cancer Research*, **44**, 2698.

MANIATIS, T., FRITSCH, E.F. & SAMBROOK, J. (1982). *Molecular Cloning: A Laboratory Manual*, Cold Spring Harbor Laboratory: New York.

MANNERVIK, B., ALIN, P., GUTHENBERG, C. & 4 others (1985). Identification of three classes of cytosolic glutathione transferases common to several mammalian species: Correlation between structural data and enzymatic properties. *Proc. Natl. Acad. Sci. USA.*, **82**, 7202.

MOSCOW, J.A., FAIRCHILD, C.R., MADDEN, M.J. & 7 others (1989). Expression of anionic glutathione-S-transferase and P-glycoprotein genes in human tissues and tumors. *Cancer Res.*, **49**, 1422.

MCQUAID, S., MCCANN, S., DAY, P., LAWLOR, E. & HUMPHRIES, P. (1989). Observations on the transcriptional activity of the glutathione S transferase gene in human haematological malignancies and in the peripheral leucocytes of cancer patients under chemotherapy. *Br. J. Cancer*, **59**, 540.

POTMESIL, M., HSIANG, Y., LIU, L. & 9 others (1988). Resistance of human leukaemic and normal lymphocytes to drug induced DNA cleavage and low levels of DNA topoisomerase II. *Cancer Res.*, **48**, 3537.

SCOTTO, K.W., BIEDLER, J.L. & MELERA, P.W. (1986). Amplification and expression of genes associated with multidrug resistance in mammalian cells. *Science*, **232**, 751.